# Supplementary material for: Affinity-guided labeling reveals P2X7 nanoscale membrane redistribution during BV2 microglial activation
Source: eLife. 2026 Jan 9;14:RP106096. doi: 10.7554/eLife.106096 (PMC12788799; doi:10.7554/eLife.106096)
Supplement: Figure 2—source data 1. [file elife-106096-fig2-data1.zip › Figure 2-source data 1.pdf]

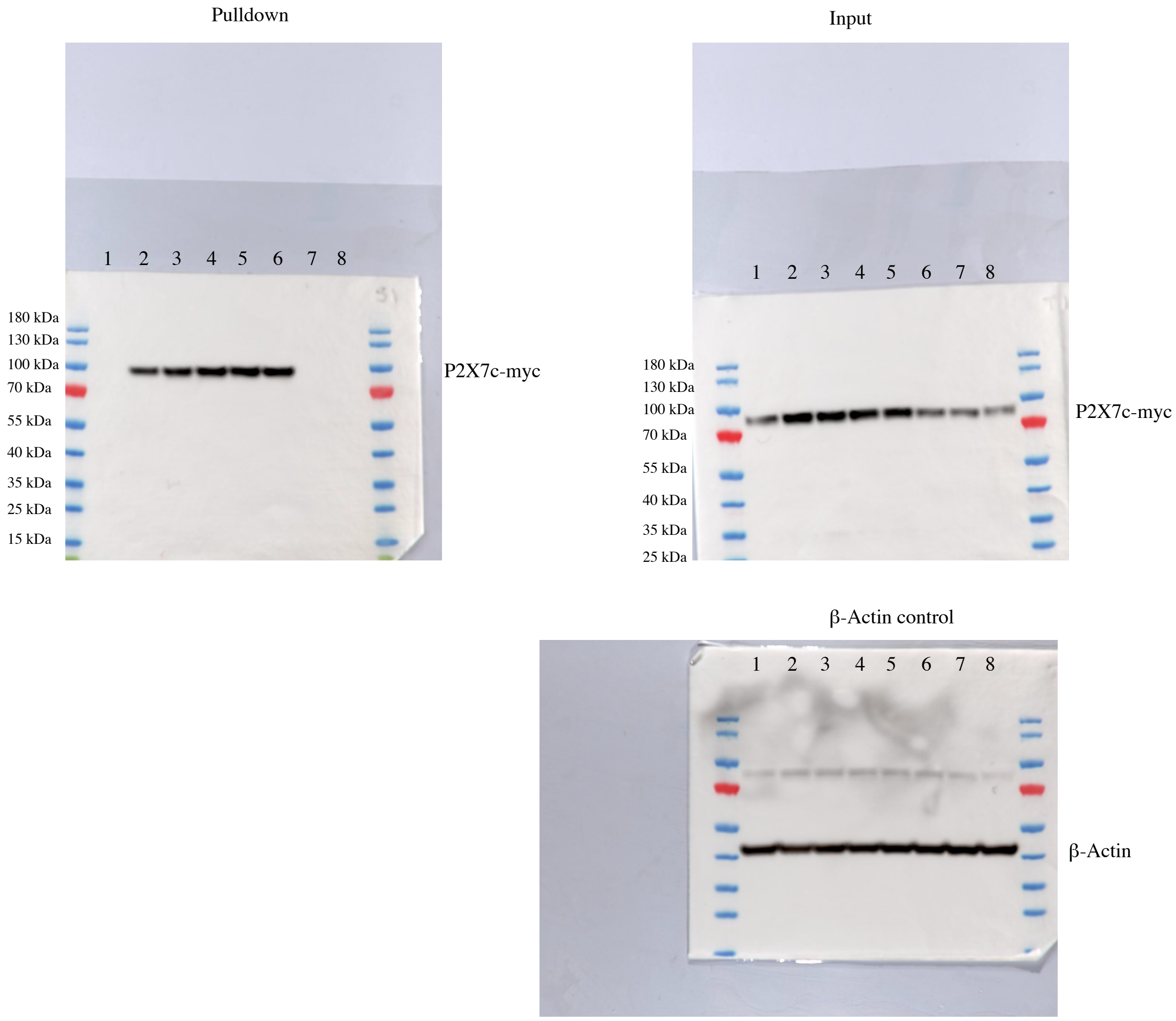

Figure 2 – source data 1. Original membranes corresponding to Figure 2C. The indicated lanes are shown in figure. Molecular weight markers are in color.
