# Supplementary figures and images for: Affinity-guided labeling reveals P2X7 nanoscale membrane redistribution during BV2 microglial activation
Source: eLife. 2026 Jan 9;14:RP106096. doi: 10.7554/eLife.106096 (PMC12788799; doi:10.7554/eLife.106096)

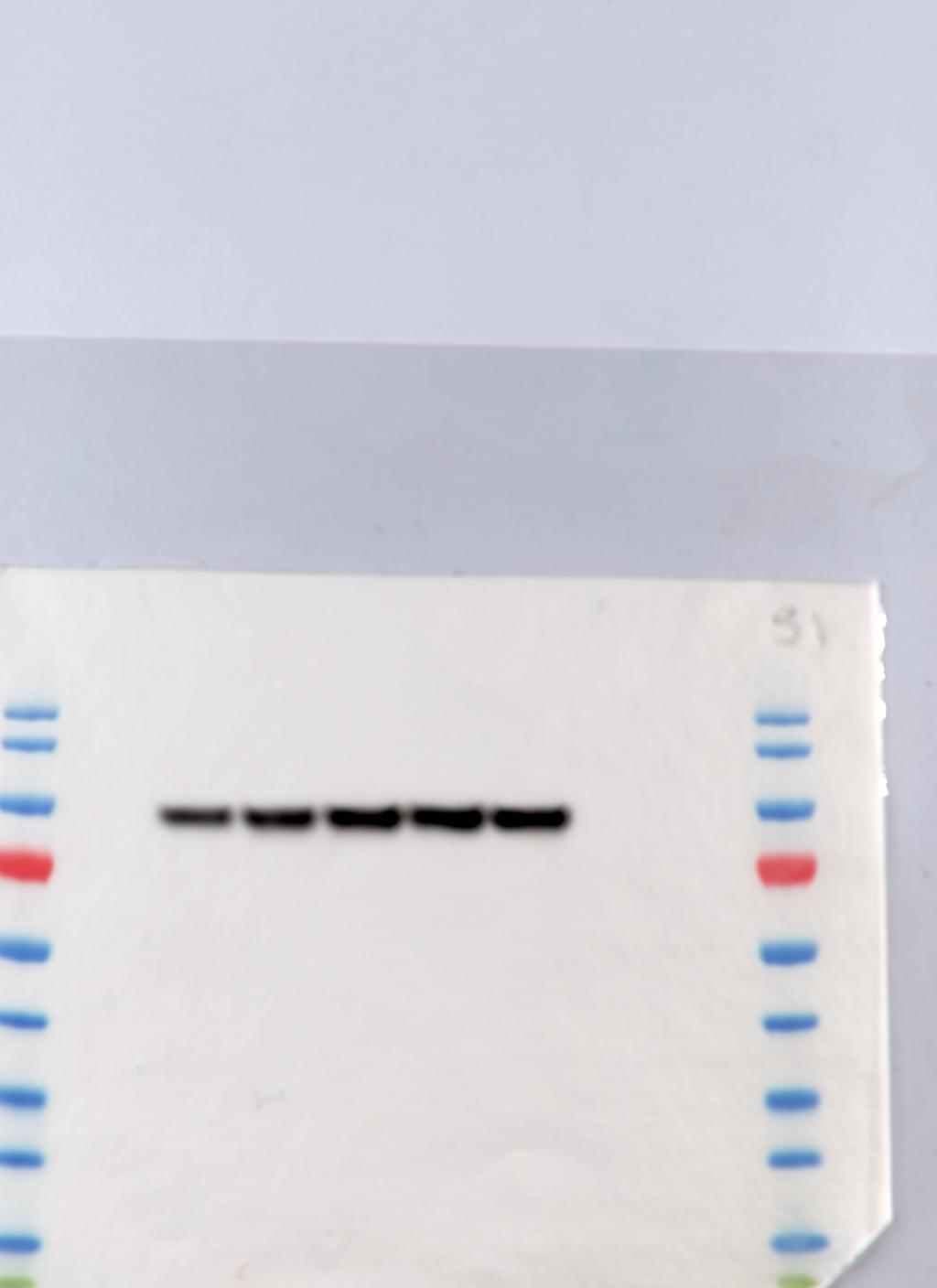

Supplement: Figure 2—source data 2. [file elife-106096-fig2-data2.zip › Figure 2-source data 2/Kinetics X7-uP pulldown.jpg]

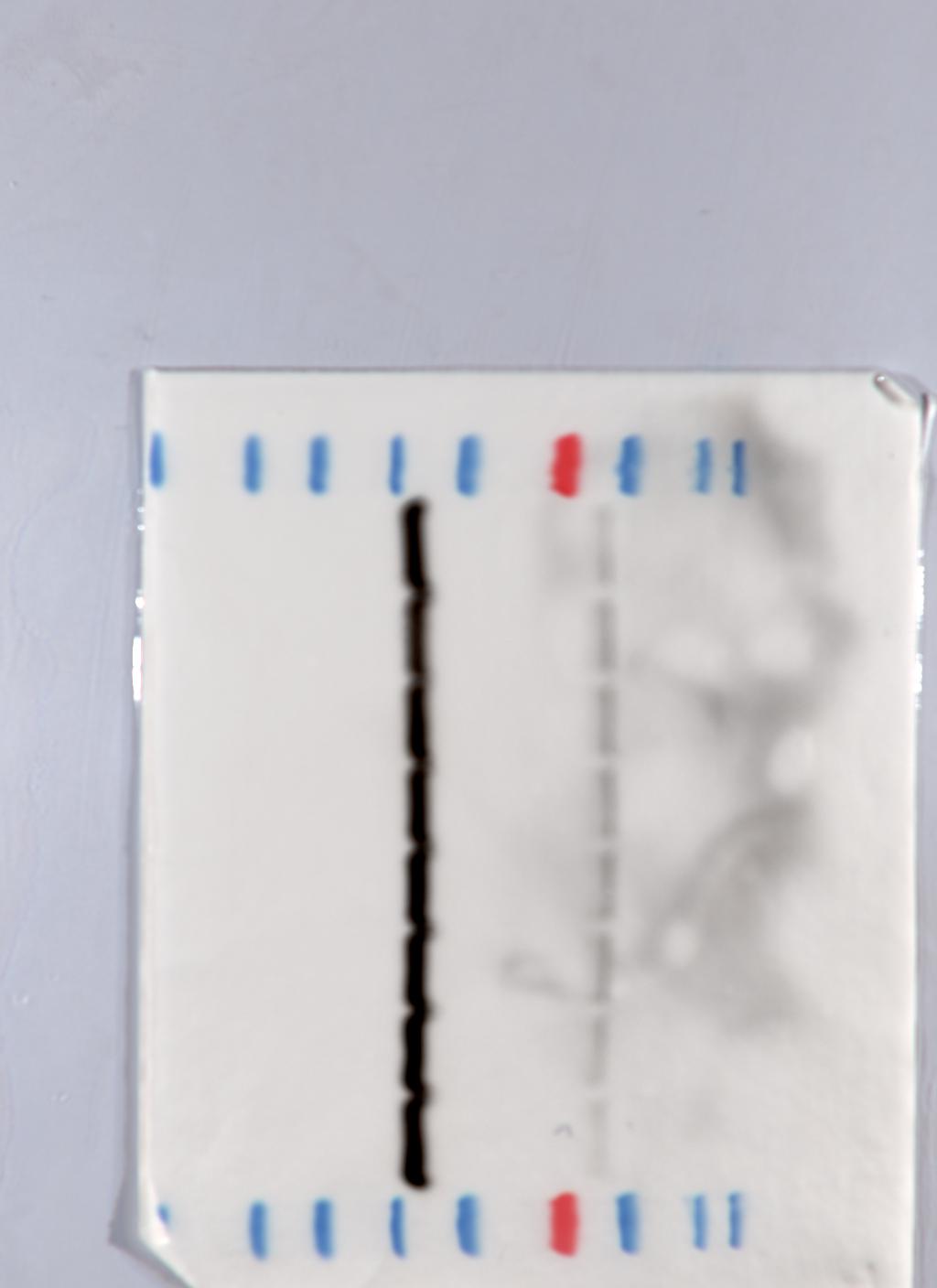

Supplement: Figure 2—source data 2. [file elife-106096-fig2-data2.zip › Figure 2-source data 2/Kinetics X7-uP control Actin.jpg]

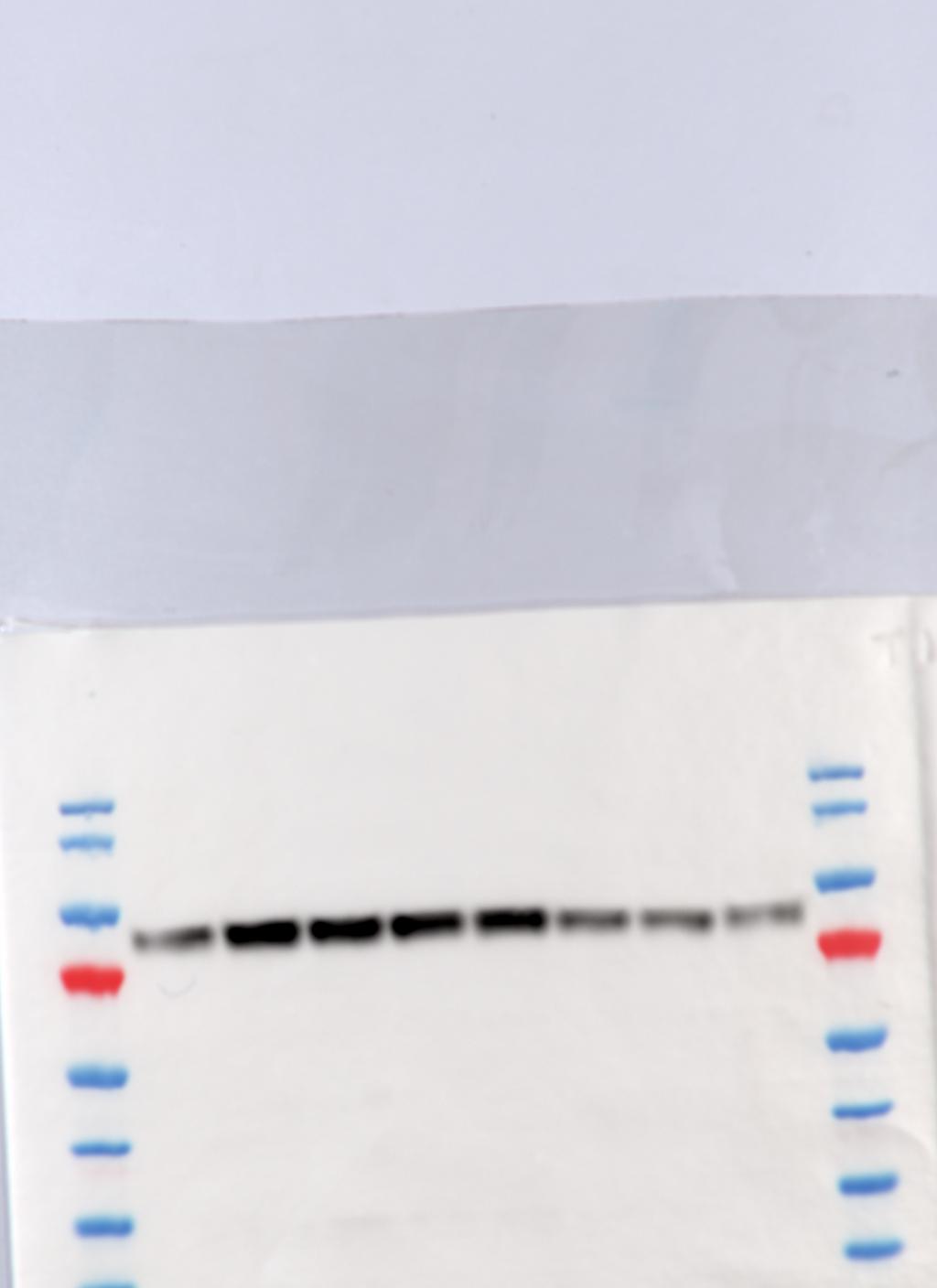

Supplement: Figure 2—source data 2. [file elife-106096-fig2-data2.zip › Figure 2-source data 2/Kinetics X7-uP input.jpg]

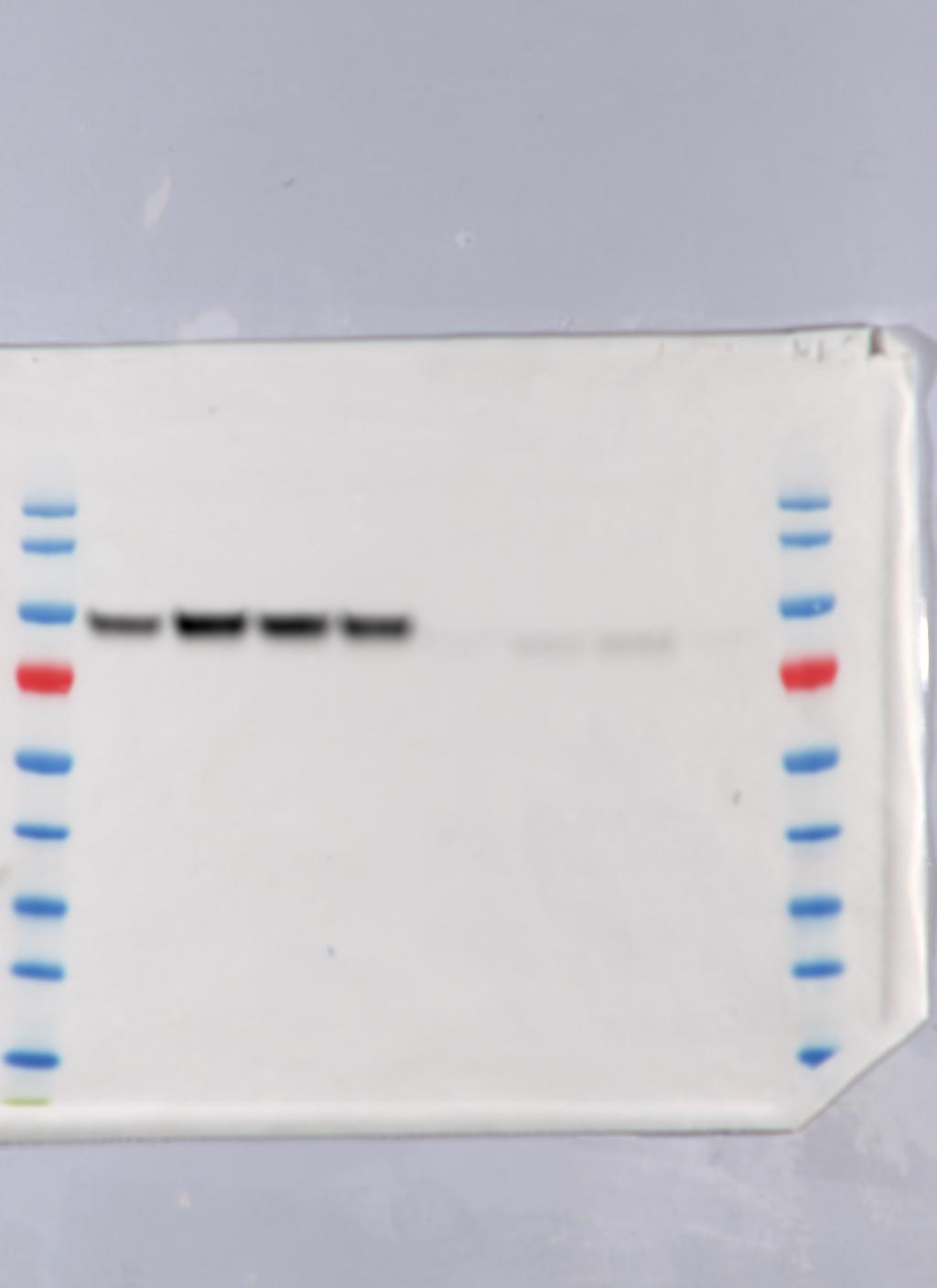

Supplement: Figure 2—figure supplement 1—source data 2. [file elife-106096-fig2-figsupp1-data2.zip › Figure 2-figure supplement 1-source data 2/Kinetics 2.5 microM X7-uP pulldown and input low exposure.png]

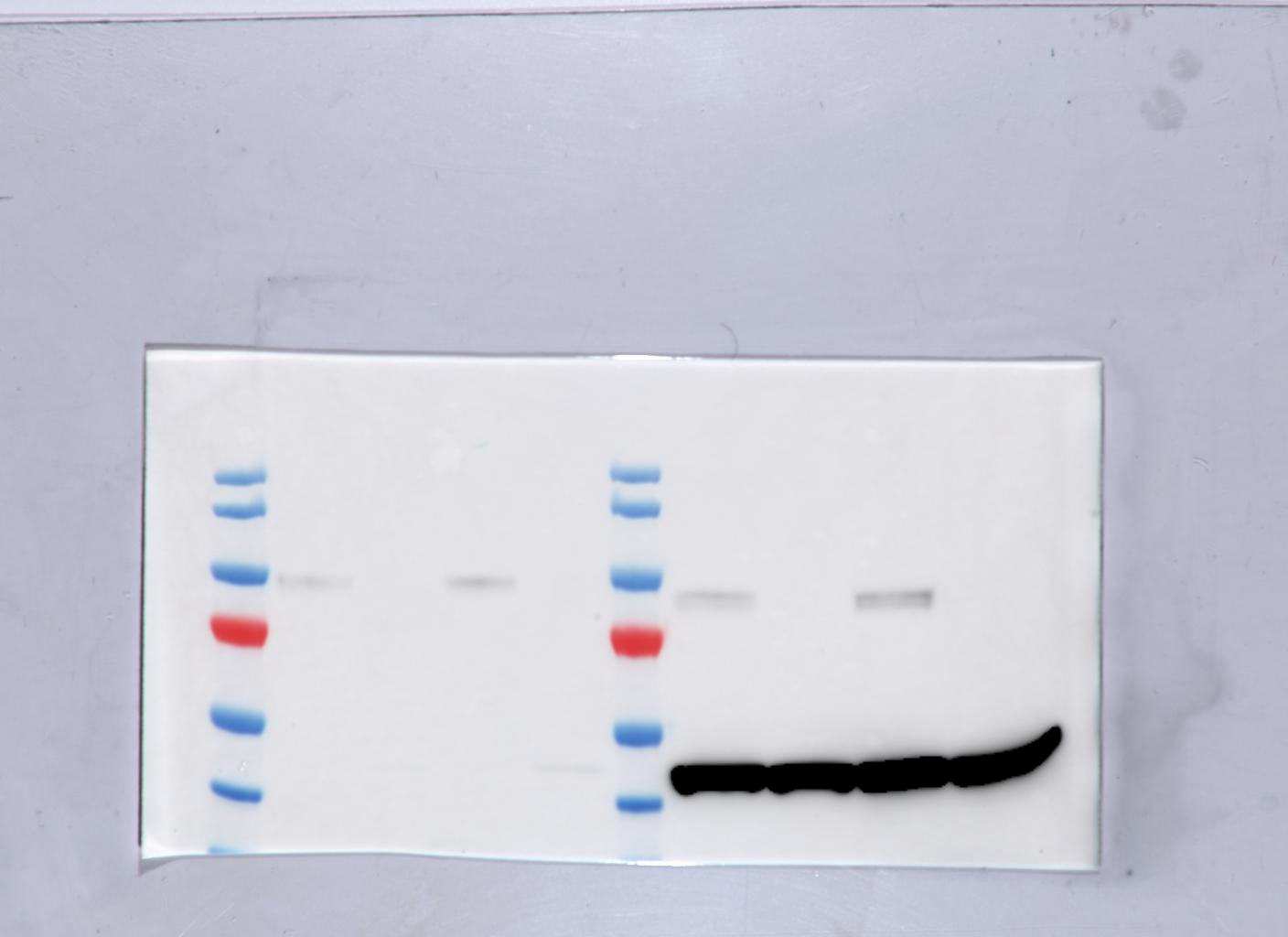

Supplement: Figure 2—figure supplement 1—source data 2. [file elife-106096-fig2-figsupp1-data2.zip › Figure 2-figure supplement 1-source data 2/P2X7c-myc pulldown, input and Actin low exposure.png]

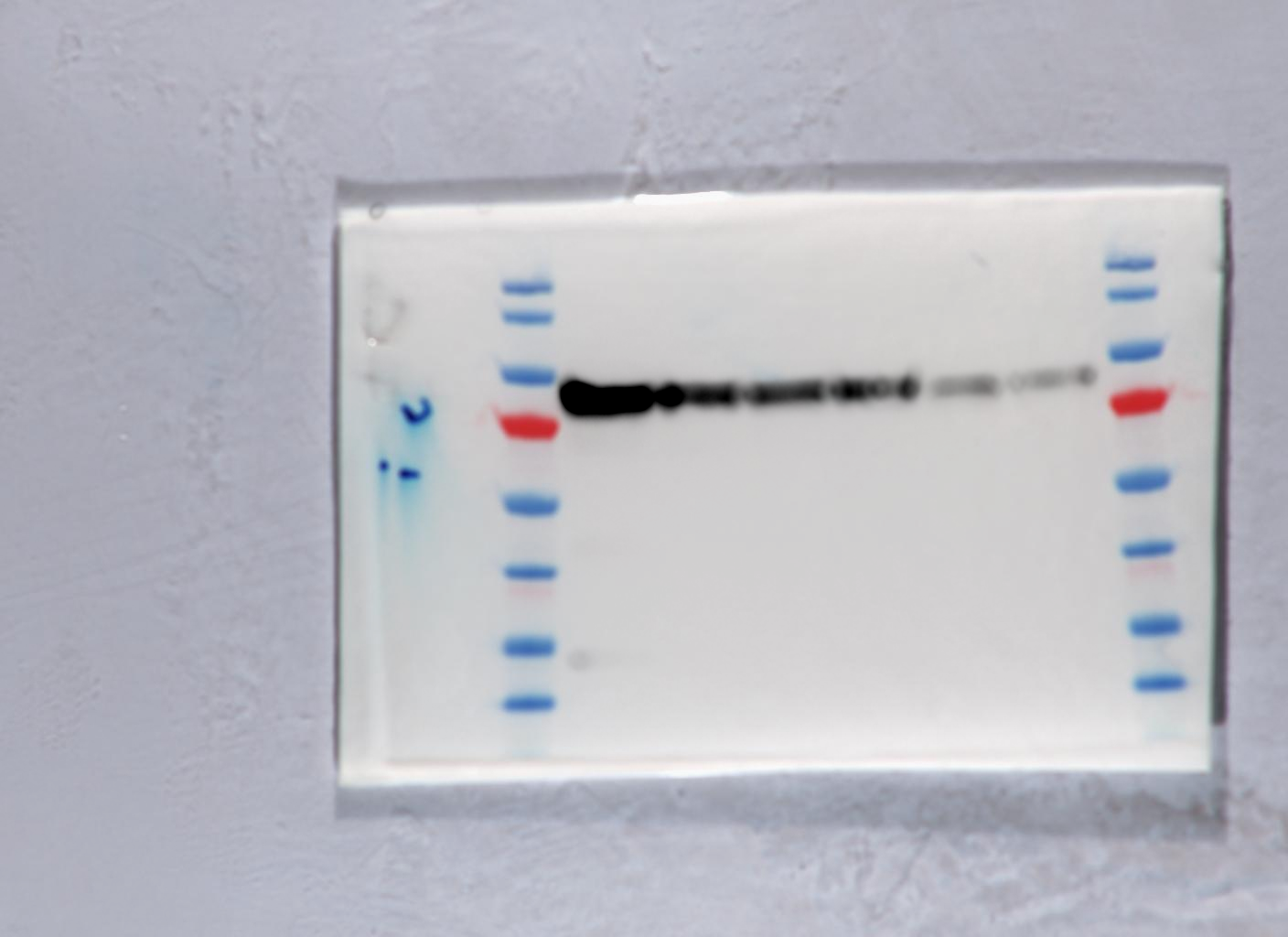

Supplement: Figure 2—figure supplement 1—source data 2. [file elife-106096-fig2-figsupp1-data2.zip › Figure 2-figure supplement 1-source data 2/Kinetics 0.5 microM X7-uP Input.png]

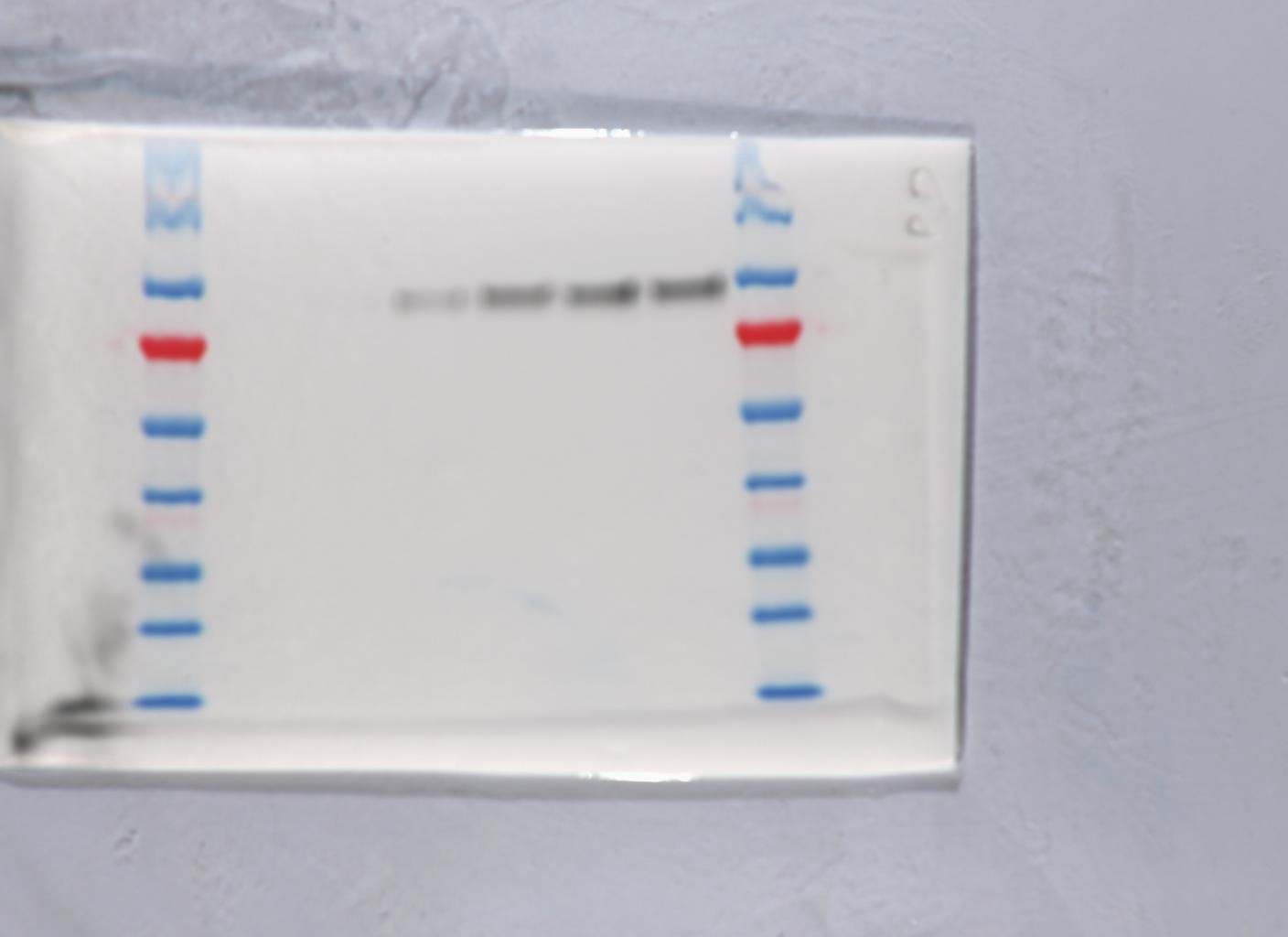

Supplement: Figure 2—figure supplement 1—source data 2. [file elife-106096-fig2-figsupp1-data2.zip › Figure 2-figure supplement 1-source data 2/Kinetics 0.5 microM X7-uP Pulldown.png]

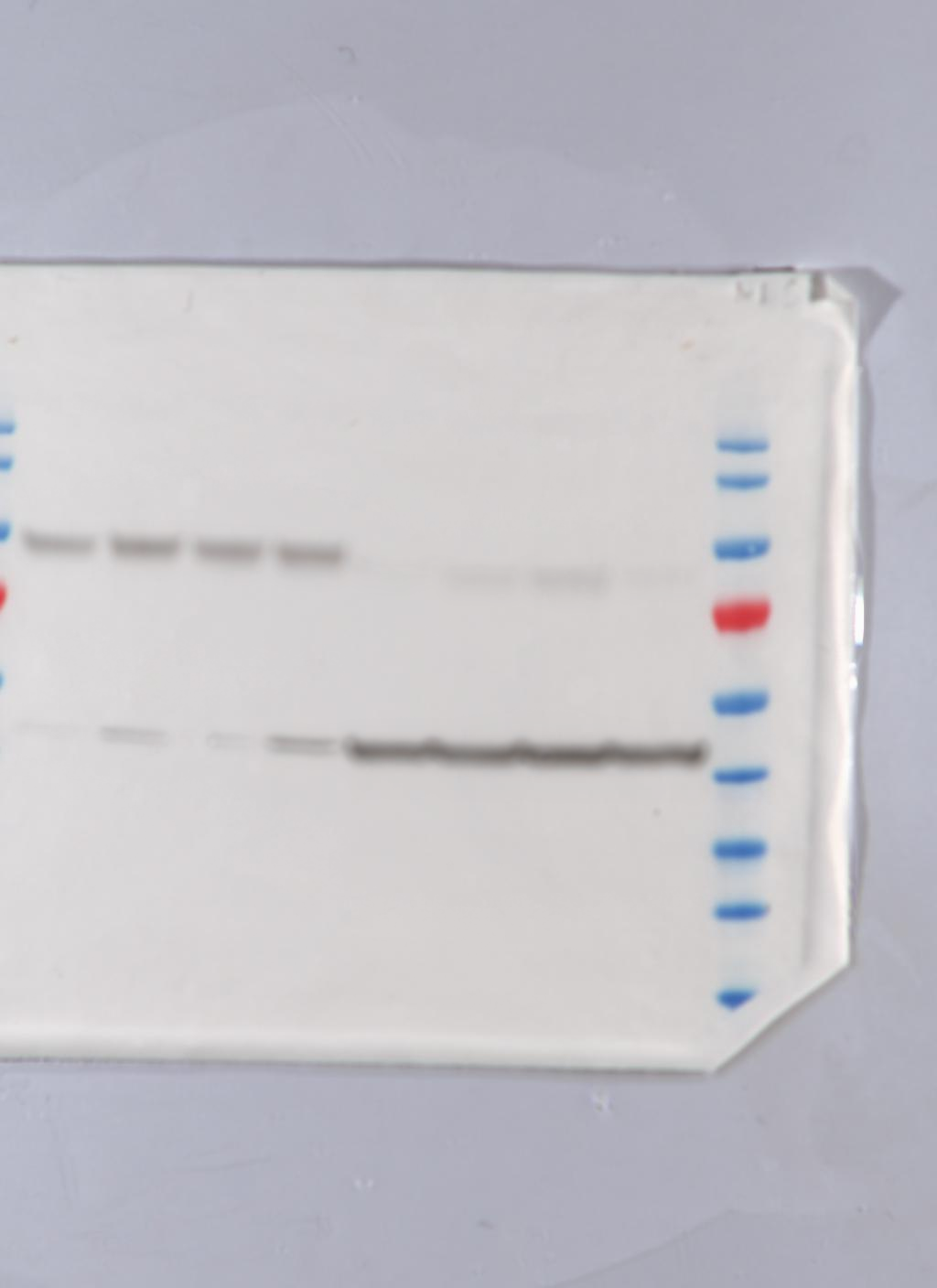

Supplement: Figure 2—figure supplement 1—source data 2. [file elife-106096-fig2-figsupp1-data2.zip › Figure 2-figure supplement 1-source data 2/kinetics 2.5 microM X7-uP Actin.png]

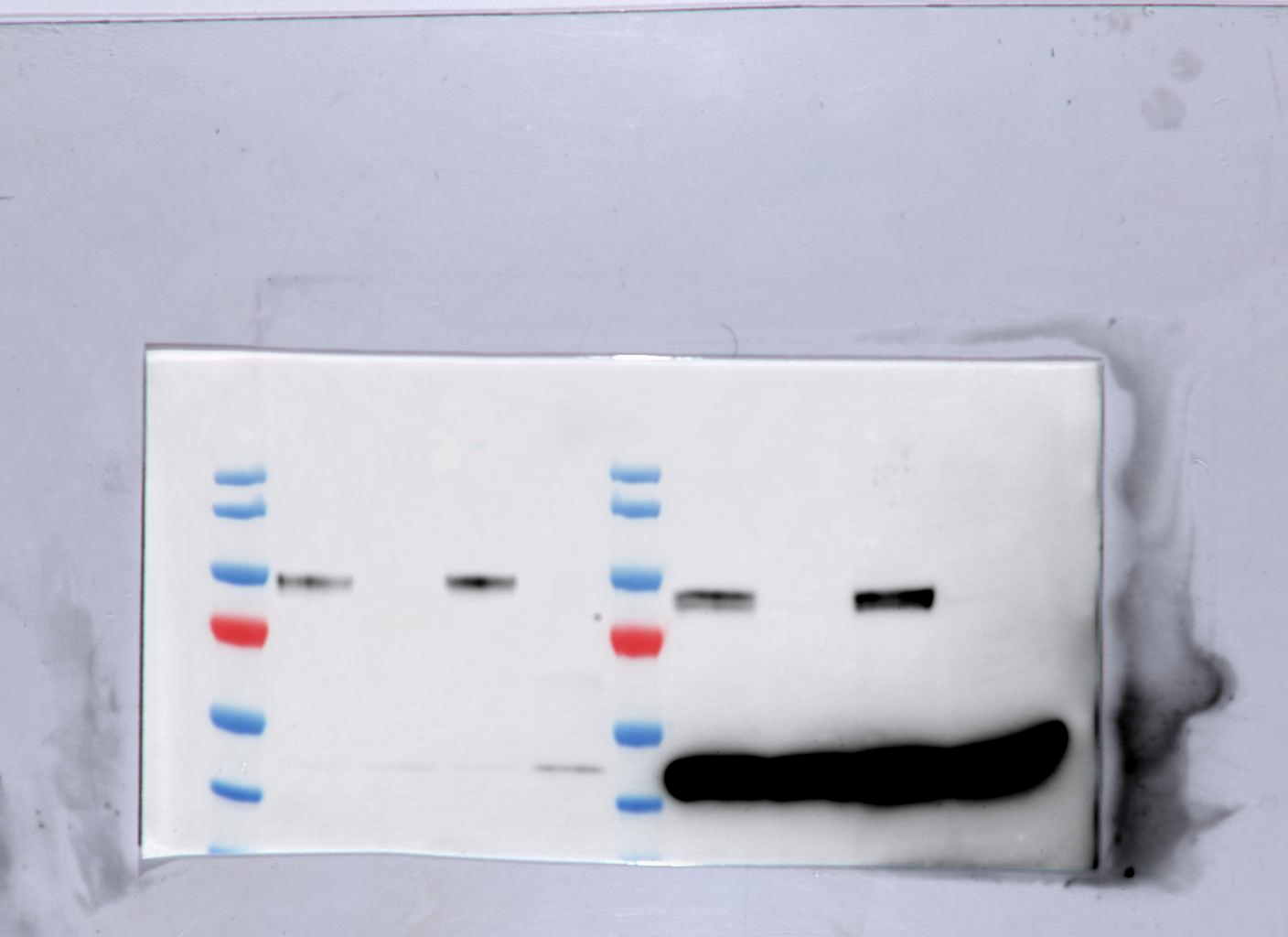

Supplement: Figure 2—figure supplement 1—source data 2. [file elife-106096-fig2-figsupp1-data2.zip › Figure 2-figure supplement 1-source data 2/P2X7c-myc pulldown, input and Actin high exposure.png]

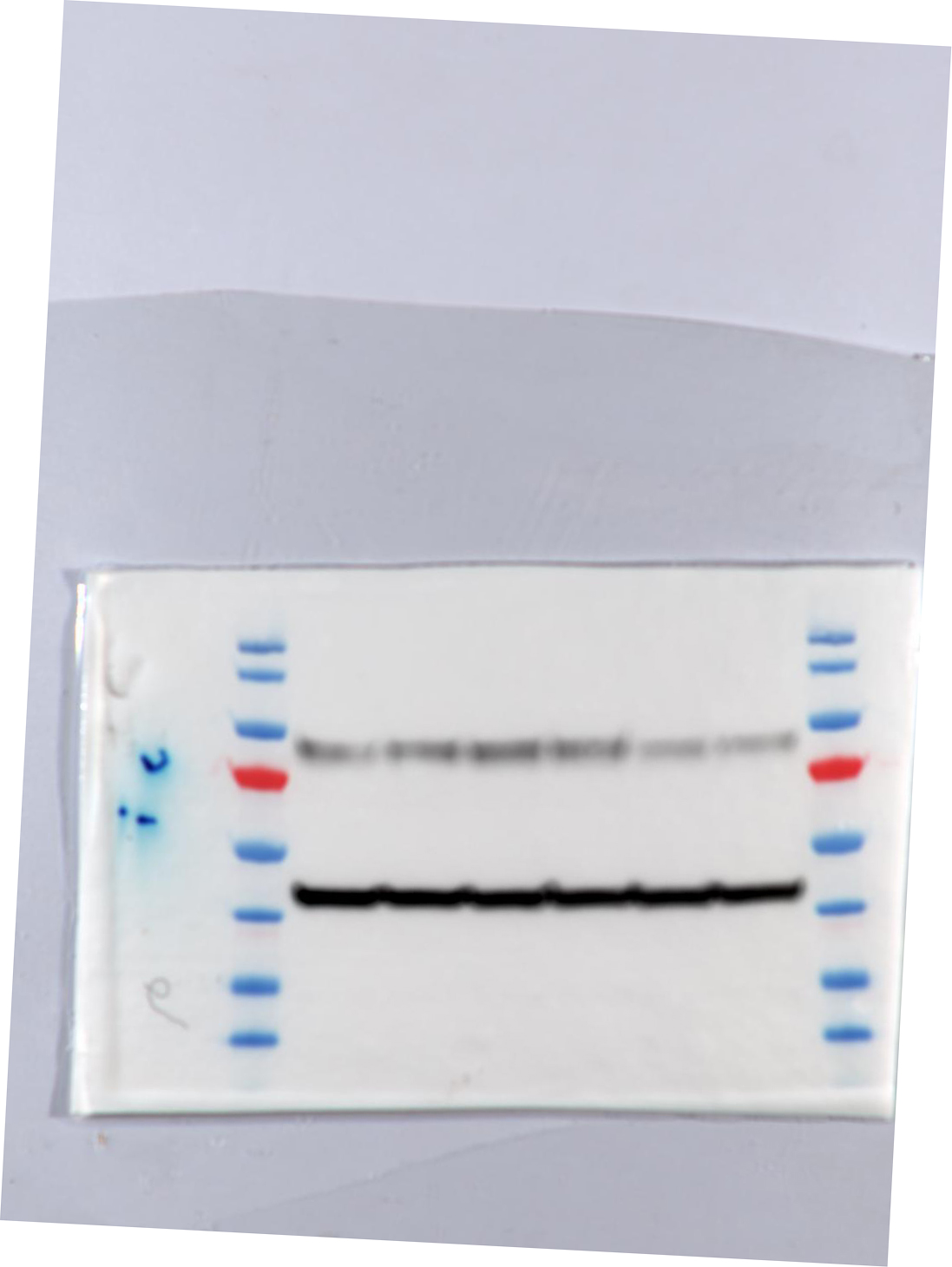

Supplement: Figure 2—figure supplement 1—source data 2. [file elife-106096-fig2-figsupp1-data2.zip › Figure 2-figure supplement 1-source data 2/Kinetics 0.5 microM X7-uP Actin.jpg]

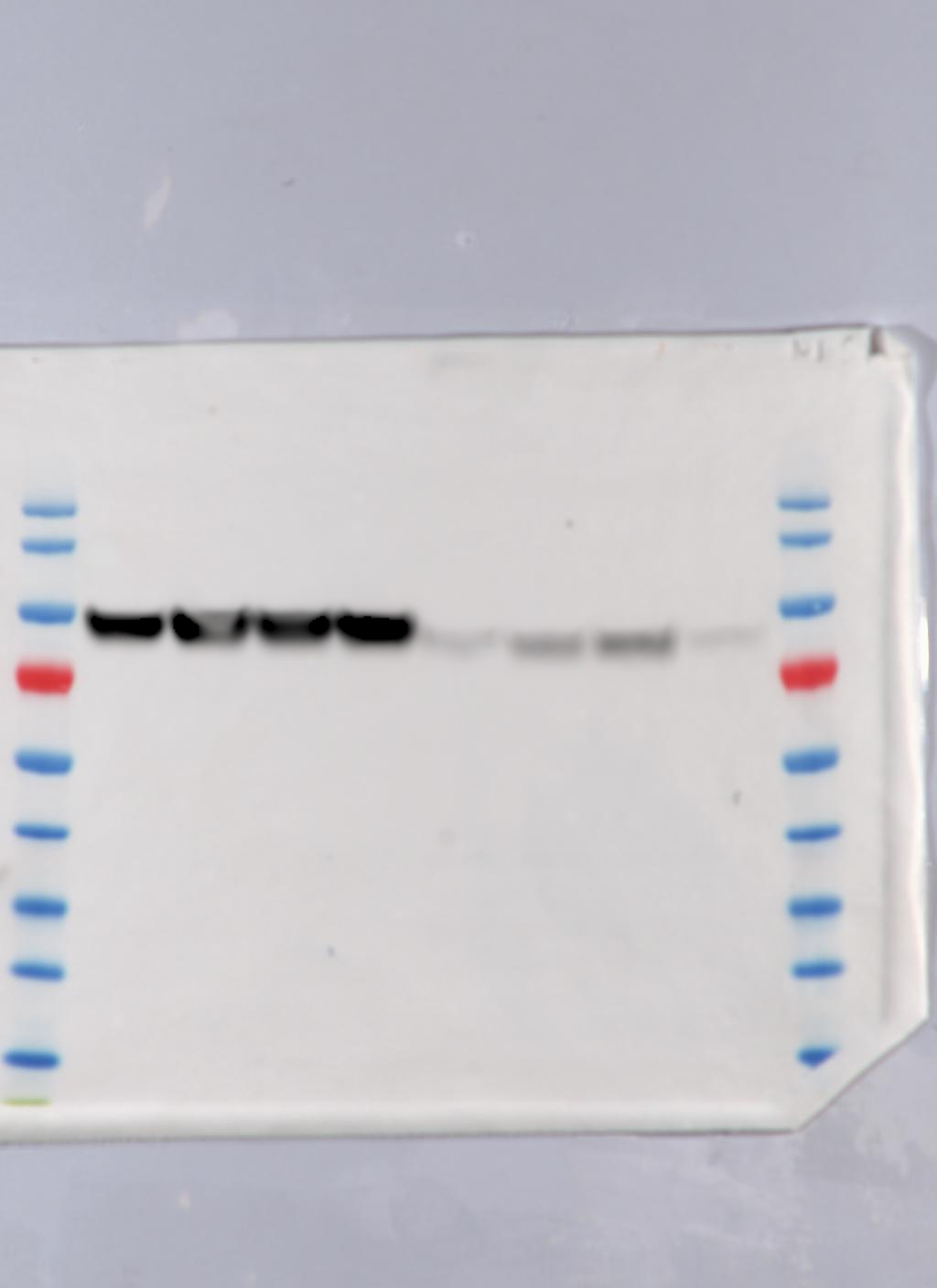

Supplement: Figure 2—figure supplement 1—source data 2. [file elife-106096-fig2-figsupp1-data2.zip › Figure 2-figure supplement 1-source data 2/Kinetics 2.5 microM X7-uP pulldown and input high exposure.png]

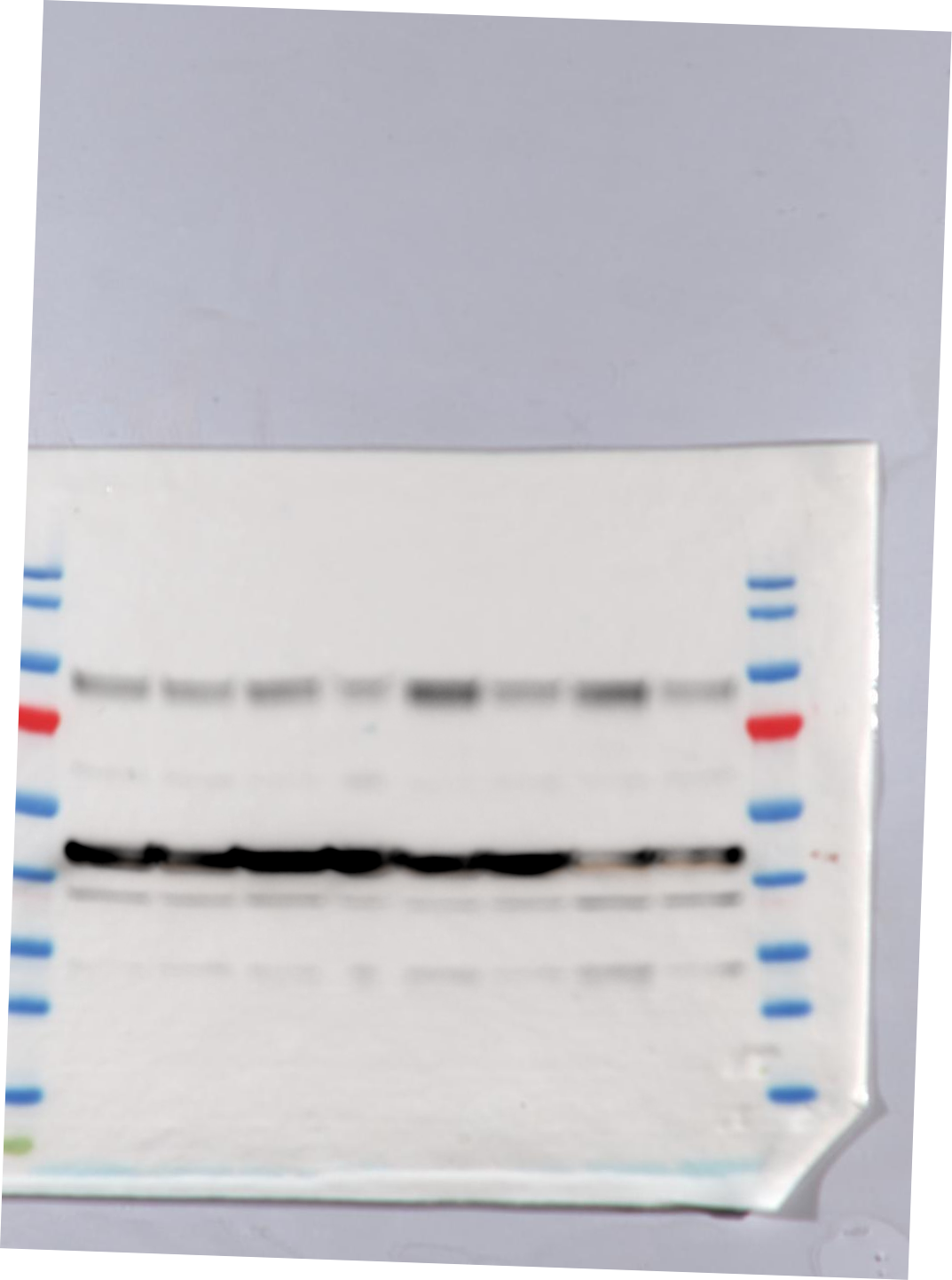

Supplement: Figure 2—figure supplement 2—source data 2. [file elife-106096-fig2-figsupp2-data2.zip › Figure 2-figure supplement 2-source data 2/Control Actin HEK cells.png]

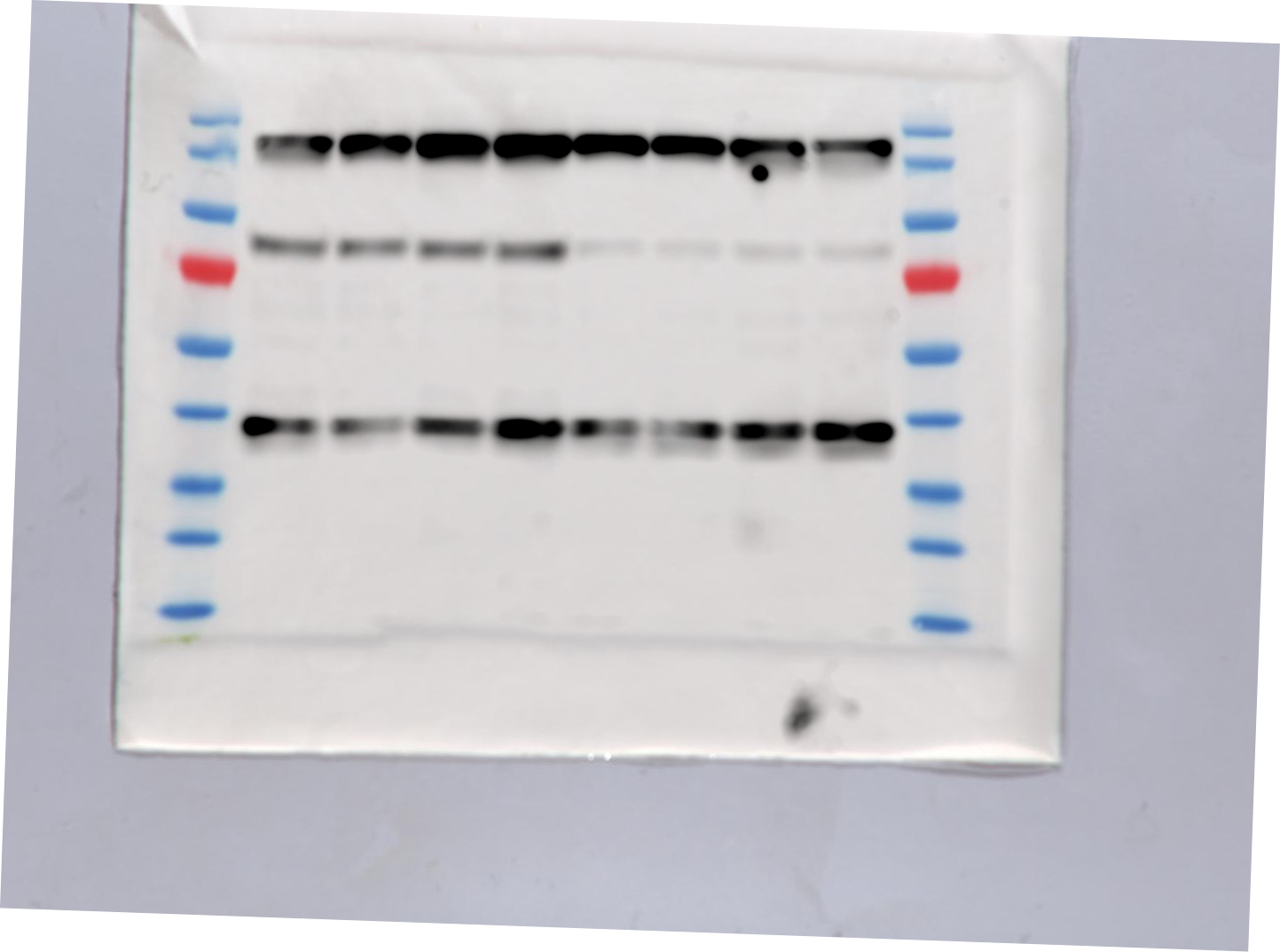

Supplement: Figure 2—figure supplement 2—source data 2. [file elife-106096-fig2-figsupp2-data2.zip › Figure 2-figure supplement 2-source data 2/X7-uP in BV2 cells.png]

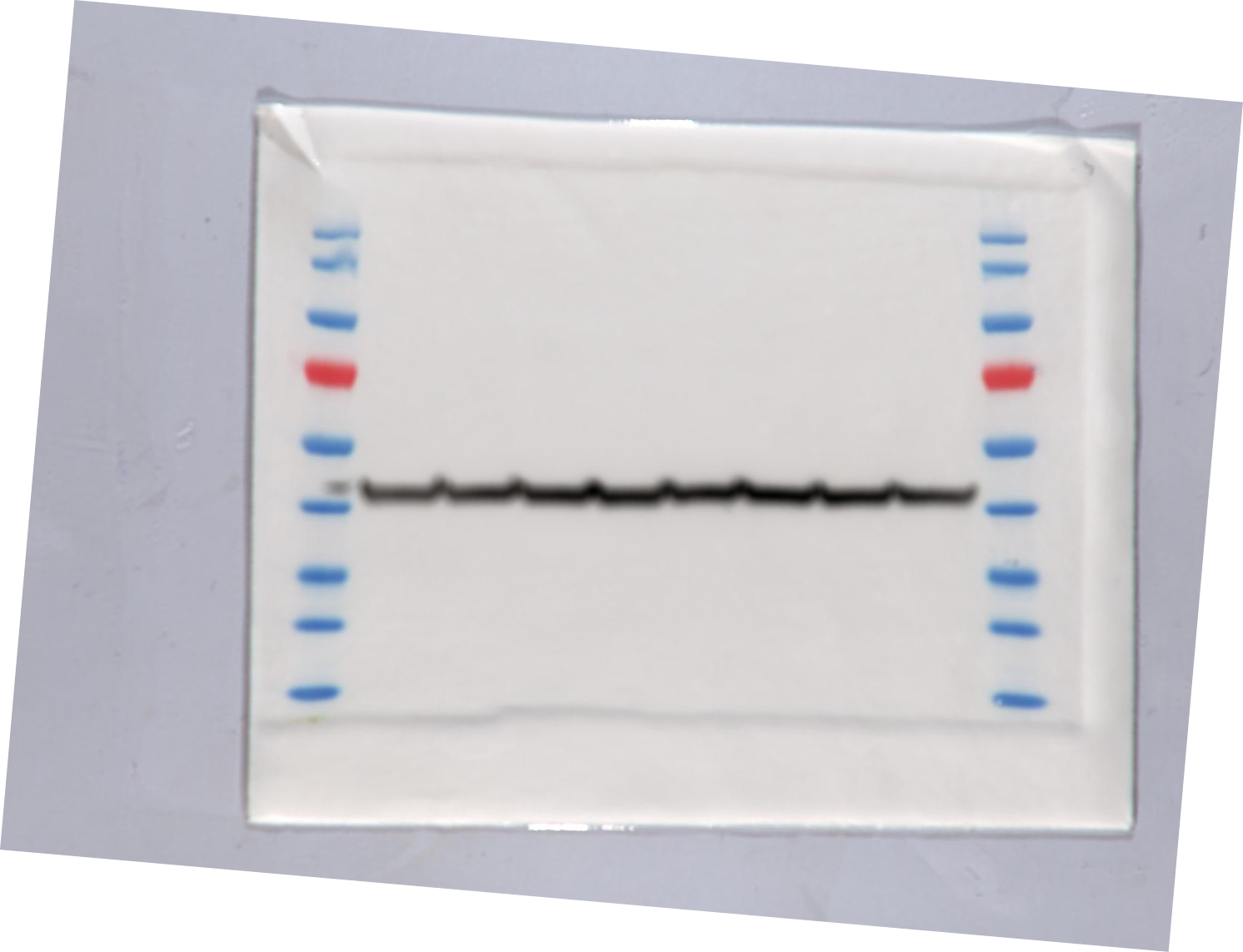

Supplement: Figure 2—figure supplement 2—source data 2. [file elife-106096-fig2-figsupp2-data2.zip › Figure 2-figure supplement 2-source data 2/Control Actin BV2 cells.png]

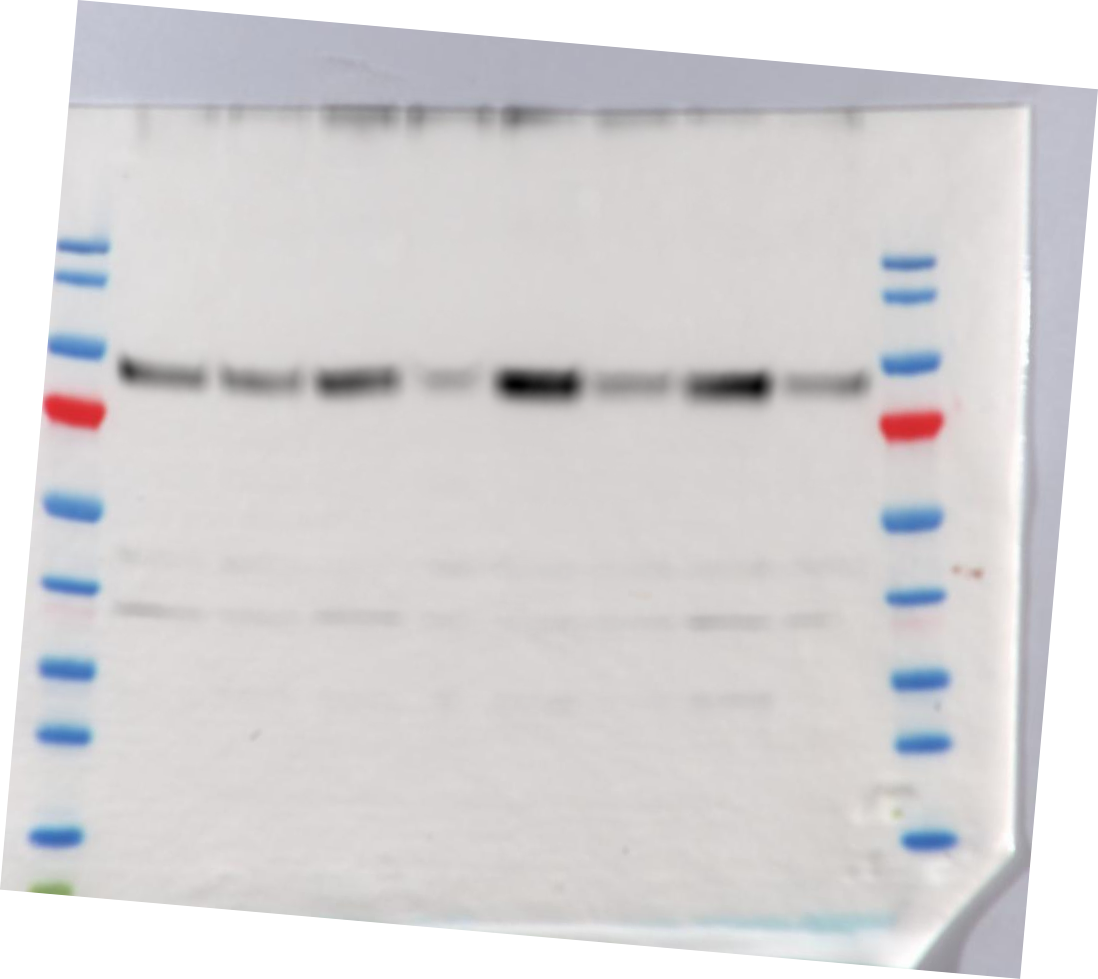

Supplement: Figure 2—figure supplement 2—source data 2. [file elife-106096-fig2-figsupp2-data2.zip › Figure 2-figure supplement 2-source data 2/X7-uP in HEK cells.png]

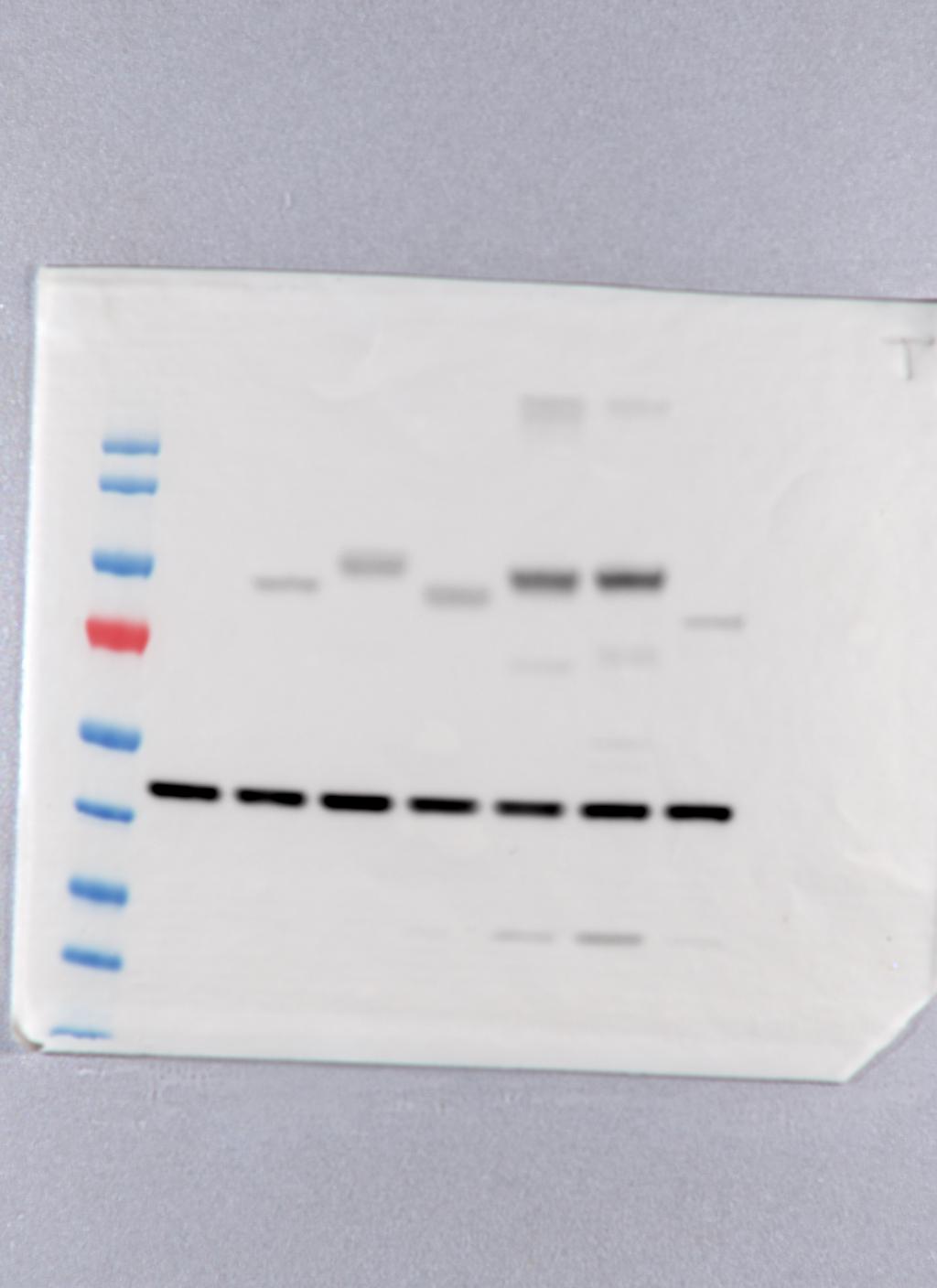

Supplement: Figure 3—figure supplement 1—source data 2. [file elife-106096-fig3-figsupp1-data2.zip › Figure 3-figure supplement 1-source data 2/Actin control for input.jpg]

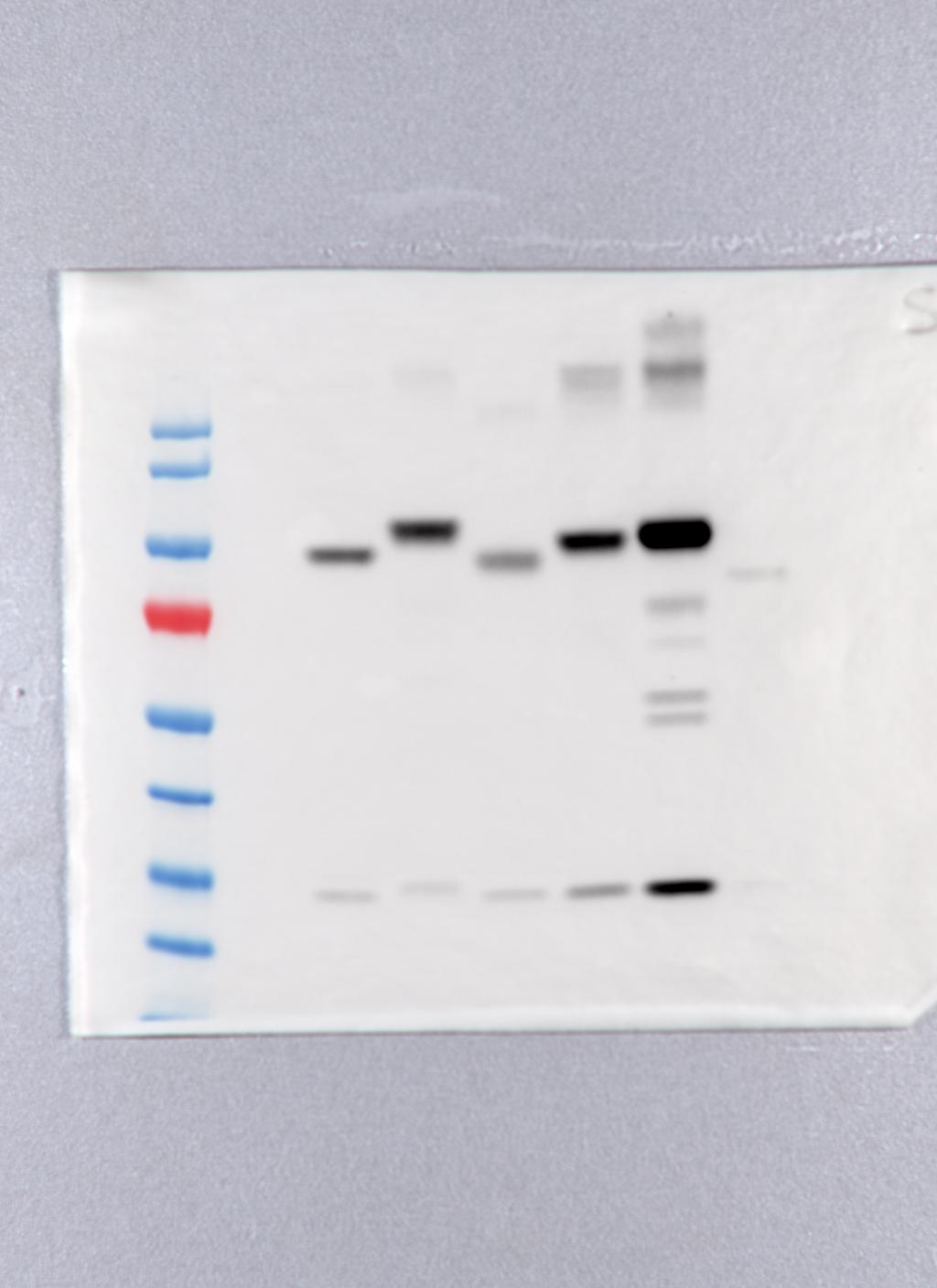

Supplement: Figure 3—figure supplement 1—source data 2. [file elife-106096-fig3-figsupp1-data2.zip › Figure 3-figure supplement 1-source data 2/Surface expression of P2X with @GFP.jpg]

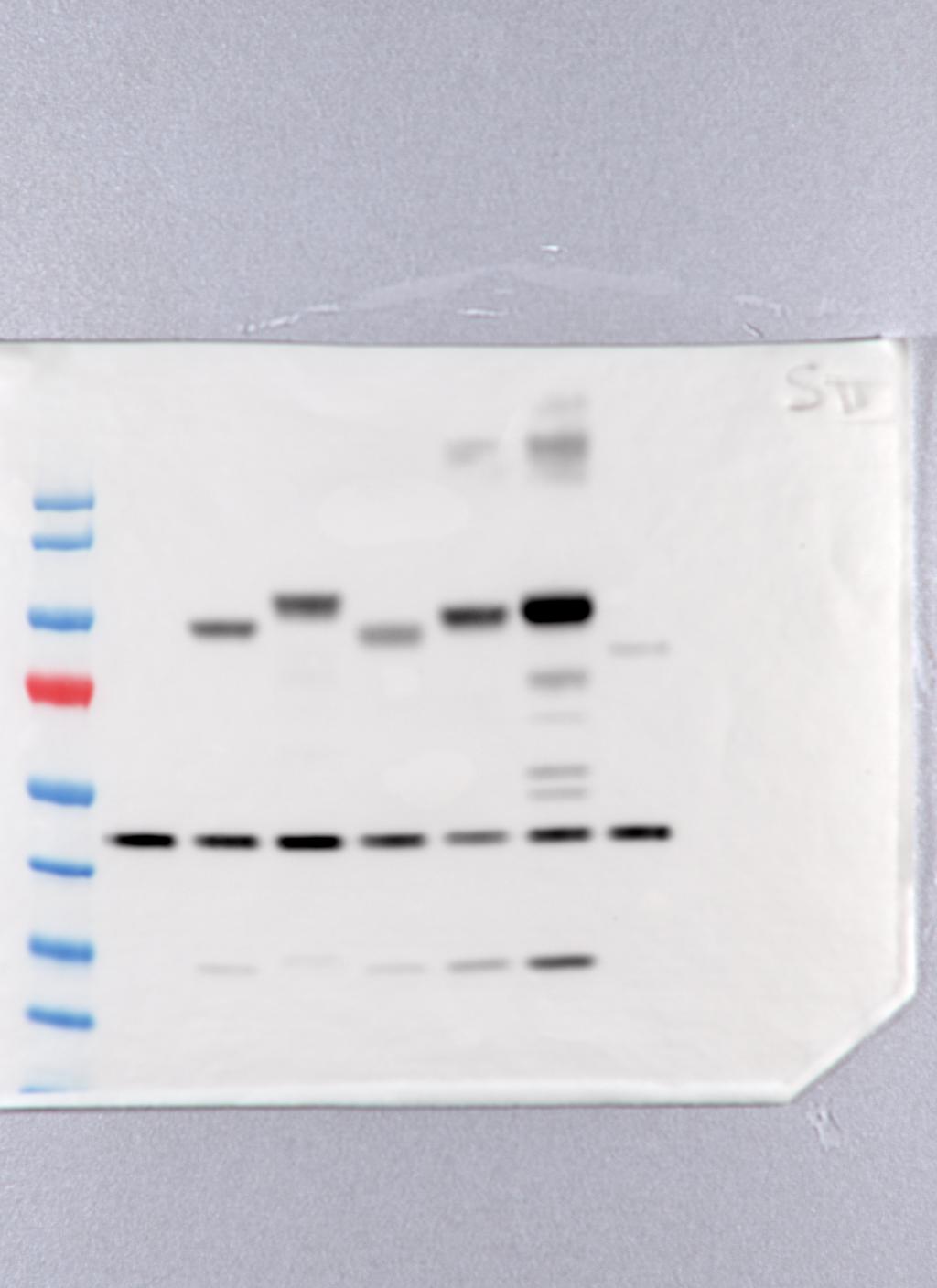

Supplement: Figure 3—figure supplement 1—source data 2. [file elife-106096-fig3-figsupp1-data2.zip › Figure 3-figure supplement 1-source data 2/Actin control for surface expression.jpg]

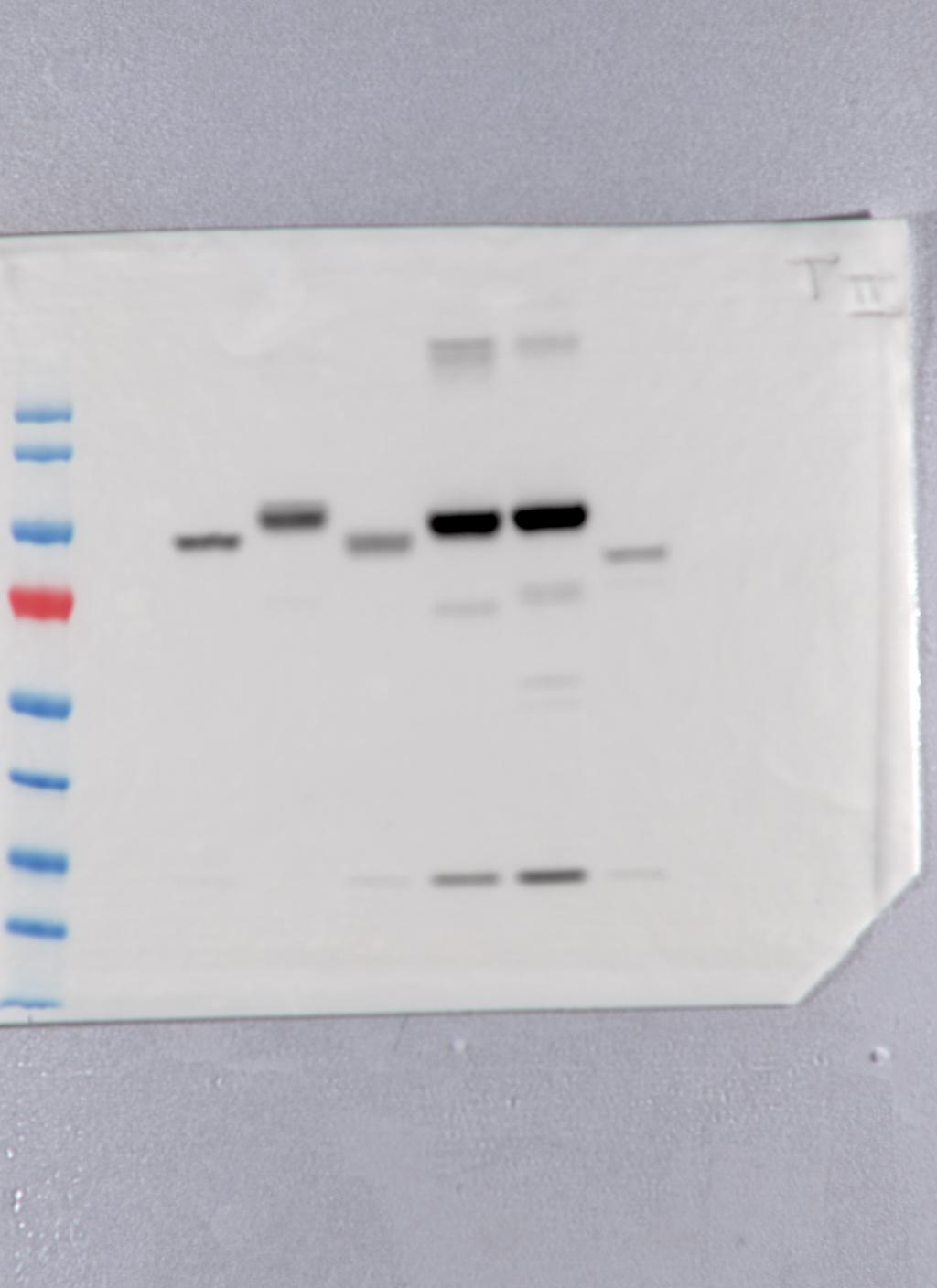

Supplement: Figure 3—figure supplement 1—source data 2. [file elife-106096-fig3-figsupp1-data2.zip › Figure 3-figure supplement 1-source data 2/Input with @GFP.jpg]

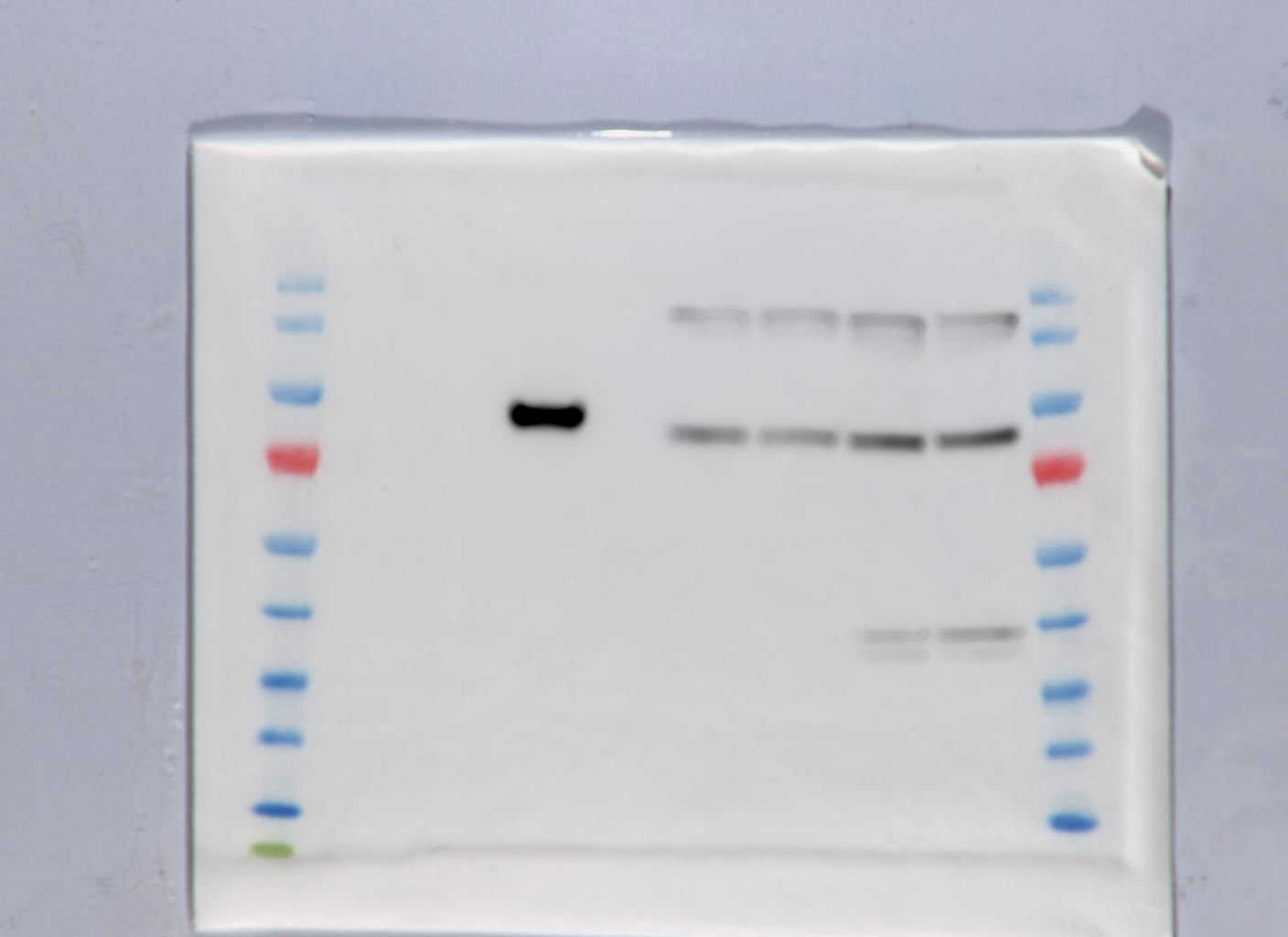

Supplement: Figure 5—figure supplement 1—source data 2. [file elife-106096-fig5-figsupp1-data2.zip › Figure 5-figure supplement 1-source data 2/X7-uP labeling in BV2 cells pulldown and input.png]

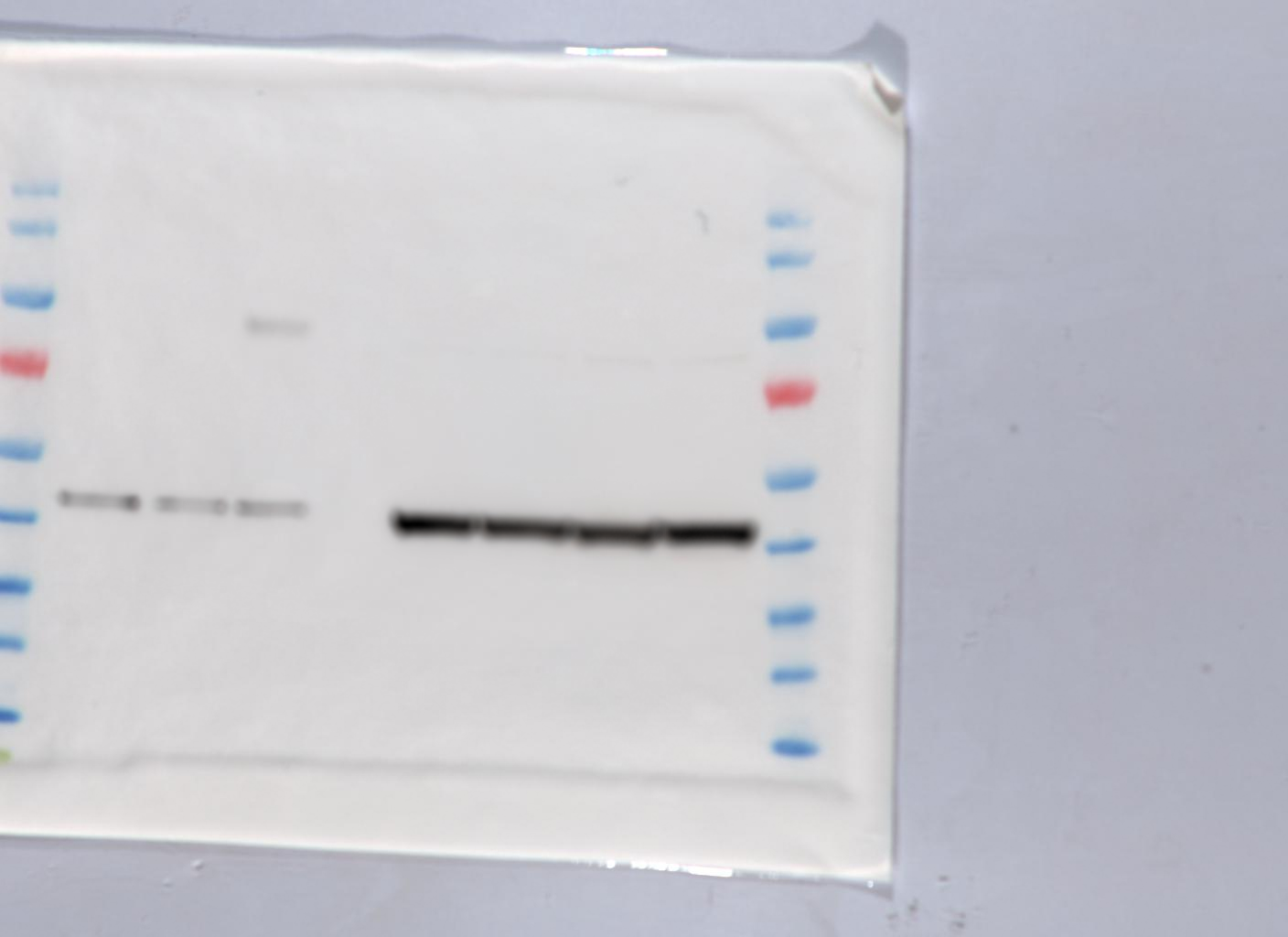

Supplement: Figure 5—figure supplement 1—source data 2. [file elife-106096-fig5-figsupp1-data2.zip › Figure 5-figure supplement 1-source data 2/X7-uP labeling in BV2 cells control Actin.png]
