# Supplementary material for: Affinity-guided labeling reveals P2X7 nanoscale membrane redistribution during BV2 microglial activation
Source: eLife. 2026 Jan 9;14:RP106096. doi: 10.7554/eLife.106096 (PMC12788799; doi:10.7554/eLife.106096)
Supplement: Figure 2—figure supplement 1—source data 1. [file elife-106096-fig2-figsupp1-data1.zip › Figure 2-figure supplement 1-source data 1.pdf]

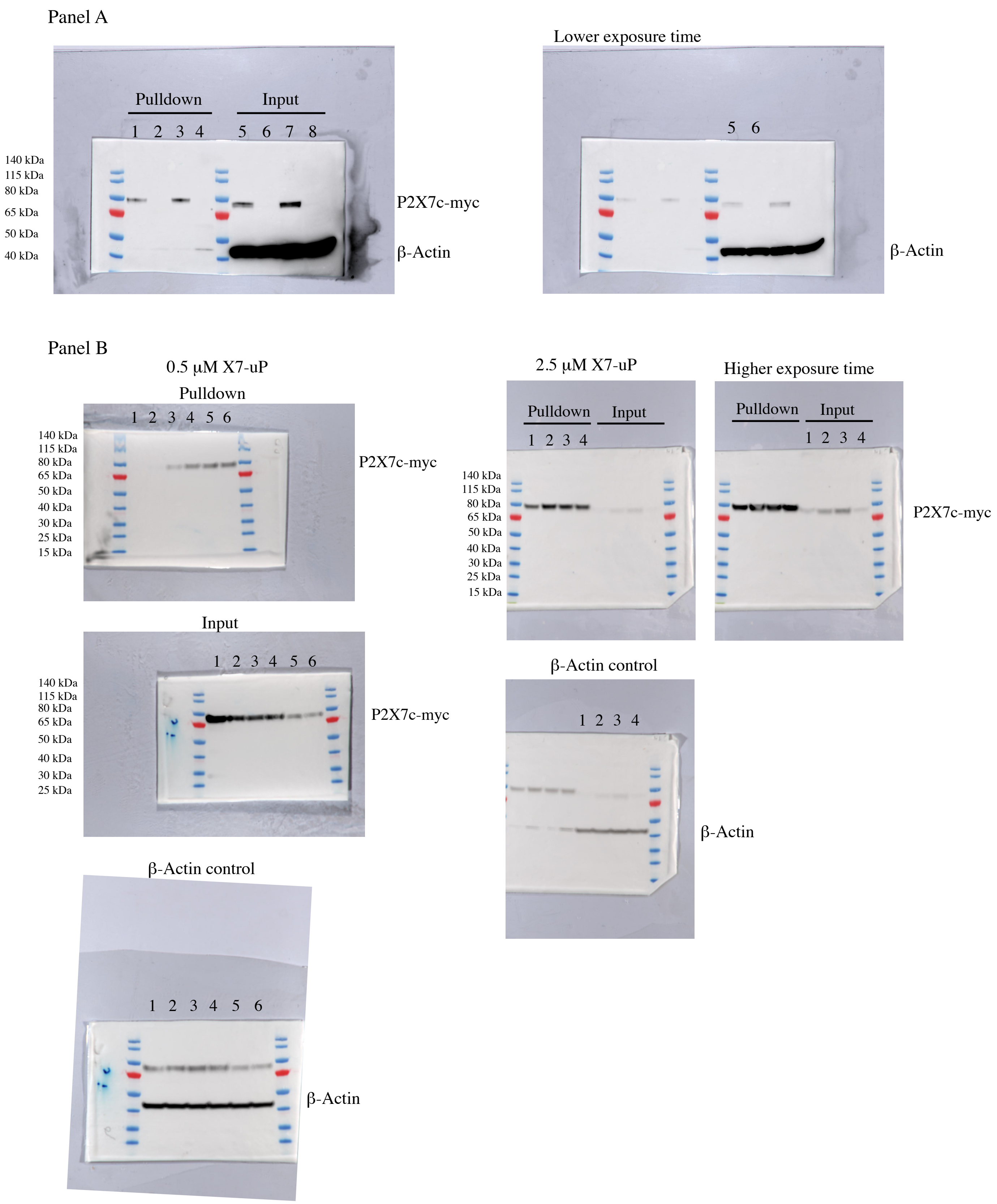

Figure 2–figure supplement 1 – source data 1. Original membranes corresponding to Figure 2–figure supplement 1A and 2B, with some membranes shown at different chemiluminescence exposure times. Lanes 1, 2, 5, and 6 in panel A are shown in Figure 2-supplement 1A, whereas lanes 3, 4, 7, and 8 are duplicates from the same experiment (not shown in the figure supplement). The β-actin control shown in Figure 2-supplement 1A is taken from lanes 5 and 6 on the membrane with lower exposure. The indicated lanes in panel B are shown in Figure 2-supplement 1B. The β-actin controls shown in Figure 2-supplement 1B are taken from lanes 1 to 6 (left membrane) and from lanes 1 to 4 (right membrane). Molecular weight markers are in color.
