# Supplementary material for: Affinity-guided labeling reveals P2X7 nanoscale membrane redistribution during BV2 microglial activation
Source: eLife. 2026 Jan 9;14:RP106096. doi: 10.7554/eLife.106096 (PMC12788799; doi:10.7554/eLife.106096)
Supplement: Figure 2—figure supplement 2—source data 1. [file elife-106096-fig2-figsupp2-data1.zip › Figure 2-figure supplement 2-source data 1.pdf]

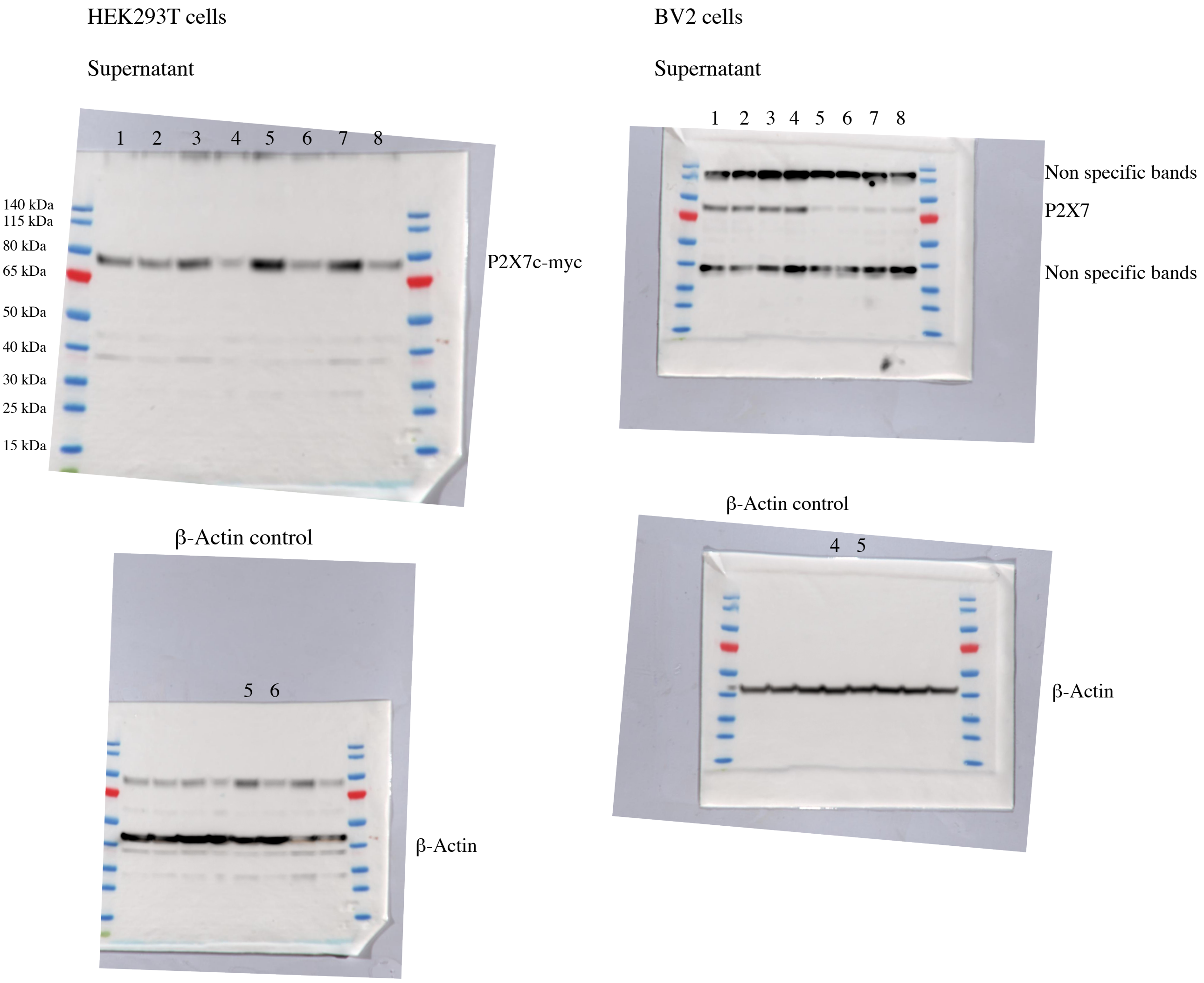

Figure 2–figure supplement 2 – source data 1. Original membranes corresponding to Figure 2–figure supplement 2A. For HEK293T cells, lanes 5 and 6 are shown in the figure supplement, whereas lanes 3, 4, 7, and 8 are duplicates (not shown in the figure supplement). Lanes 1 and 2 contain other samples. Specifically, lanes 3, 5, and 7 correspond to untreated cells, and lanes 4, 6, and 8 correspond to X7-uP–treated HEK cells. For BV2 cells, lanes 4 and 5 are shown in the figure supplement, whereas the remaining lanes are duplicates. Specifically, lanes 1 to 4 correspond to untreated BV2 cells, and lanes 5 to 8 to X7-uP–treated BV2 cells. The β-actin controls shown in figure supplement are taken from lanes 5 and 6 for HEK cells and from lanes 4 and 5 for BV2 cells. Molecular weight markers are in color.
