# Supplementary material for: Affinity-guided labeling reveals P2X7 nanoscale membrane redistribution during BV2 microglial activation
Source: eLife. 2026 Jan 9;14:RP106096. doi: 10.7554/eLife.106096 (PMC12788799; doi:10.7554/eLife.106096)
Supplement: Figure 3—figure supplement 1—source data 1. [file elife-106096-fig3-figsupp1-data1.zip › Figure 3-figure supplement 1-source data 1.pdf]

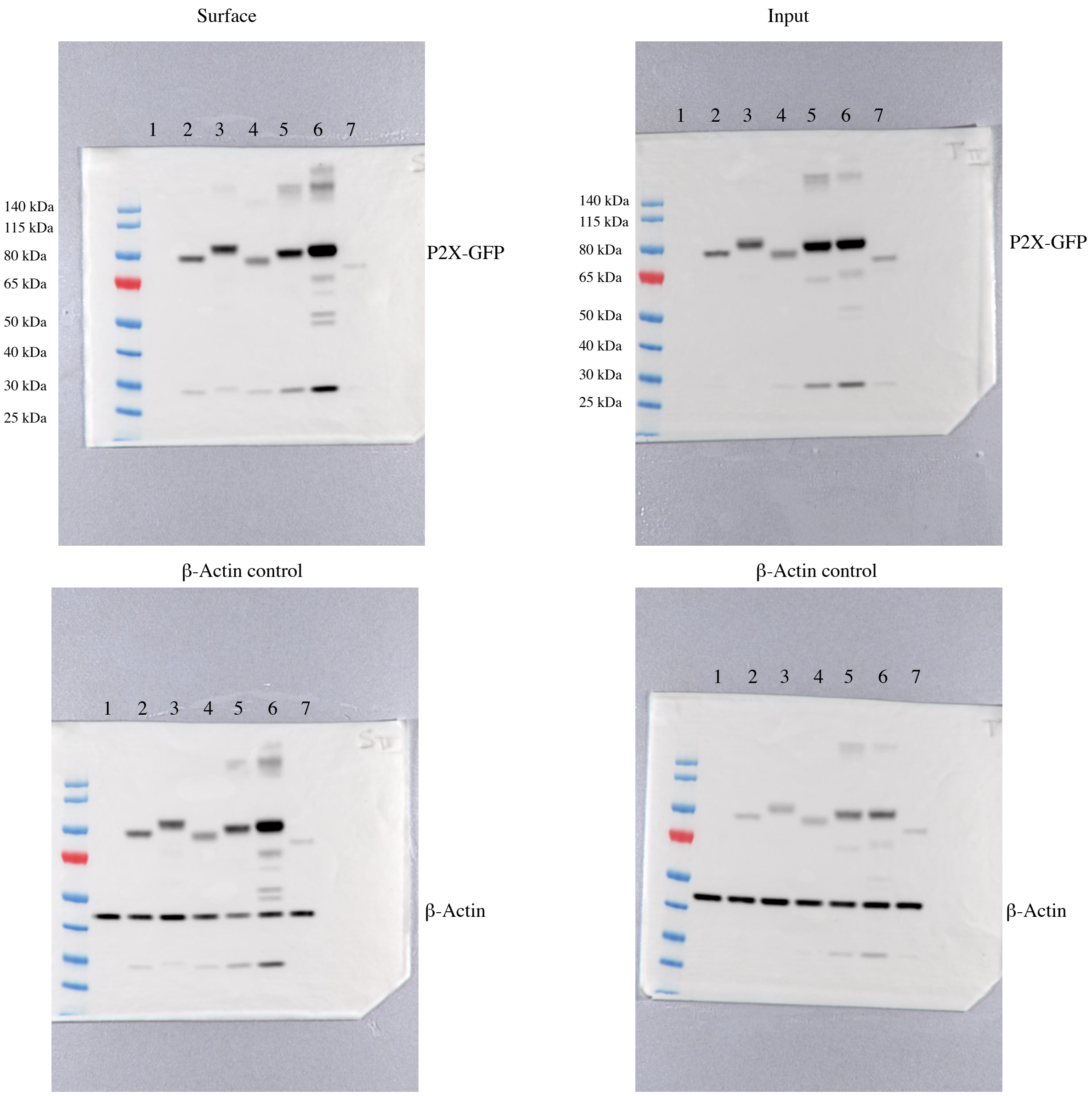

Figure 3–figure supplement 1 – source data 1. Original membranes corresponding to Figure 3–figure supplement 1A and 1B. The indicated lanes are shown in figure. Molecular weight markers are in color.
